# Toxicological and Medical Aspects of *Aspergillus*-Derived Mycotoxins Entering the Feed and Food Chain

**DOI:** 10.3389/fmicb.2019.02908

**Published:** 2020-01-09

**Authors:** Zsolt Ráduly, László Szabó, Anett Madar, István Pócsi, László Csernoch

**Affiliations:** ^1^Department of Physiology, Faculty of Medicine, University of Debrecen, Debrecen, Hungary; ^2^Doctoral School of Molecular Medicine, University of Debrecen, Debrecen, Hungary; ^3^Department of Molecular Biotechnology and Microbiology, Faculty of Science and Technology, Institute of Biotechnology, University of Debrecen, Debrecen, Hungary

**Keywords:** mycotoxin, aflatoxins, ochratoxins, fumonisins, sterigmatocystin, food poisoning, carcinogenic, secondary metabolites

## Abstract

Due to Earth’s changing climate, the ongoing and foreseeable spreading of mycotoxigenic *Aspergillu*s species has increased the possibility of mycotoxin contamination in the feed and food production chain. These harmful mycotoxins have aroused serious health and economic problems since their first appearance. The most potent *Aspergillus*-derived mycotoxins include aflatoxins, ochratoxins, gliotoxin, fumonisins, sterigmatocystin, and patulin. Some of them can be found in dairy products, mainly in milk and cheese, as well as in fresh and especially in dried fruits and vegetables, in nut products, typically in groundnuts, in oil seeds, in coffee beans, in different grain products, like rice, wheat, barley, rye, and frequently in maize and, furthermore, even in the liver of livestock fed by mycotoxin-contaminated forage. Though the mycotoxins present in the feed and food chain are well documented, the human physiological effects of mycotoxin exposure are not yet fully understood. It is known that mycotoxins have nephrotoxic, genotoxic, teratogenic, carcinogenic, and cytotoxic properties and, as a consequence, these toxins may cause liver carcinomas, renal dysfunctions, and also immunosuppressed states. The deleterious physiological effects of mycotoxins on humans are still a first-priority question. In food production and also in the case of acute and chronic poisoning, there are possibilities to set suitable food safety measures into operation to minimize the effects of mycotoxin contaminations. On the other hand, preventive actions are always better, due to the multivariate nature of mycotoxin exposures. In this review, the occurrence and toxicological features of major *Aspergillus*-derived mycotoxins are summarized and, furthermore, the possibilities of treatments in the medical practice to heal the deleterious consequences of acute and/or chronic exposures are presented.

## Introduction

Each mycotoxin is a secondary metabolite produced by fungi, but not all secondary metabolites are toxic (Bennett and Klich, 2003; Richard, 2007). Apart from mycotoxins, other secondary metabolites are often produced by fungi, e.g., plant growth regulators, pharmaceutically useful compounds, and pigments (Richard, 2007). These biological compounds usually play a part in the survival of fungi and, concomitantly, are disadvantageous for their surroundings as well (Bennett and Klich, 2003; Keller, 2019). Various types of environmental stress may trigger the production of these deleterious compounds, suggesting their protective role, e.g., under oxidative stress (Reverberi et al., 2010). Hence, the production of mycotoxins may facilitate the successful adaptation of fungi to a broad spectrum of environmental stress conditions (Schmidt-Heydt et al., 2009), which are raised, e.g., by the changing environment and climate (Van der Fels-Klerx and Camenzuli, 2016; Medina et al., 2017). Mycotoxin production may help fungi in competition with other microorganisms (Hymery et al., 2014) or to resist against grazing by insects (Rohlfs, 2015). In host – phytopathogenic fungus interactions, mycotoxins may inhibit the germination of seeds and may also contribute to the invasion of plant tissues *via* eliciting versatile apoptotic and necrotic cell death processes (Pusztahelyi et al., 2015).

Since 1962, when almost 100,000 turkeys died in an unusual veterinary crisis in London, the field of mycotoxin research has become a relevant scientific issue. That particular “turkey X disease” was linked to peanut meals, which were contaminated by aflatoxins (Sweeney and Dobson, 1998; Smith et al., 2016). This specific new field of knowledge was called mycotoxicology, which includes all areas of research related to mycotoxins; meanwhile, the term mycotoxicosis covers all animal and human diseases caused by mycotoxins. Mycotoxins can be classified according to their chemical structures, origin of biosynthesis, and characteristic symptoms assigned to the particular toxins. In this paper, we aim at summarizing the medical risks of consuming food contaminated by *Aspergillus*-derived mycotoxins. Additionally, we included a brief overview on some socioeconomic and environmental impacts of mycotoxin food and feed contaminations, possibilities for prevention, and the available decontamination methods and medical treatments.

**FIGURE 1:**
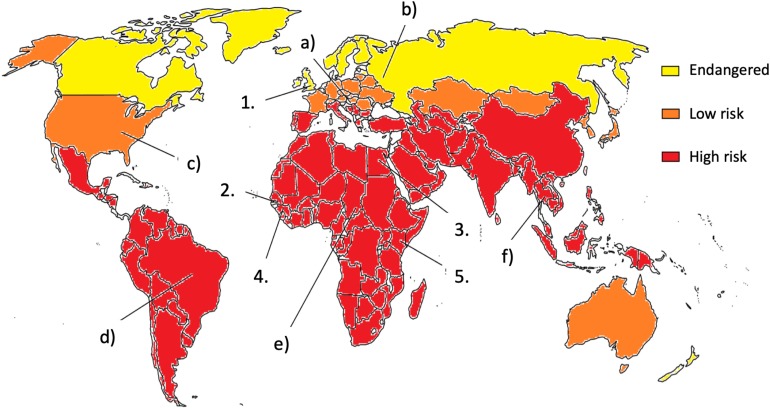
The risk of mycotoxin exposure. Milestones in mycotoxicology: (1) In 1962, mycotoxins are identified as cause of turkey “X” disease; (2) Aflatoxin outbreak in Gambia in 1988, No. subjects: 391; (3) Aflatoxin poisoning in Egypt in 1992, No. subjects: 19; **(4)** Aflatoxin outbreak in Guinea in 1999, No. subjects: approx. 600; and (5) Aflatoxin outbreak in Kenya 2004, No. subjects: approx. 100; **(a)** Due to the climate change and increasing mean temperature, mycotoxin-producing fungi spread to the north. **(b)** Monoculture farming is sensitive to mold infestation. **(c)** Strict federal regulation can prevent the spread of mold. **(d)** The strict regulation of import and export are important to minimalize mycotoxin contaminations. **(e)** Prevention of mycotoxin infestation is of primary importance. Without sufficient education and up-to-date methods, it is hard to store, process, transport, or even analyze properly and safely food and feed. **(f)** Mycotoxins have serious economic and financial consequences (see references in the text).

During the past 60 years, it has become clear that the world has to deal with mycotoxin exposure (see Figure 1). Agricultural commodities are often contaminated with mycotoxins, which results in either visible, acute effects or chronic, long-term hidden health damages (Souers et al., 2013; Smith et al., 2016; Udovicki et al., 2018; Rushing and Selim, 2019). As maize, rice, and wheat are among the most important crops, the presence of mycotoxins in these feed and foodstuffs entails a high public health risk of chronic exposure to mycotoxins (Jard et al., 2011; Rodrigues et al., 2011; Smith et al., 2016; Udovicki et al., 2018). The food shortage typical of mainly developing countries resulted in necessary negligence of the mycotoxin content of food and feed. The lack of knowledge about mycotoxins and their effects, safety regulations and enforcement, infrastructure to monitor and quantify the mycotoxin content, and the lack of political will all contribute to mycotoxin exposures. These regrettable circumstances led to the continuous risk of mycotoxin poisoning and the worsening of living conditions in the affected regions, especially in the case of children. Although mycotoxicoses mainly occur in developing regions of the world, recent years showed that industrialized countries in the moderate climate belt also have to face the risks of *Aspergillus-*derived toxin exposure (Cleveland et al., 2003; Udovicki et al., 2018). The occurrence and spread of molds depend on several factors, including environmental, social, and economic conditions (Omotayo et al., 2019). Grain producers and exporters in the world encountered the challenging problem of how mycotoxin contents in food and feed should be somehow regulated (Cleveland et al., 2003; EC 1881/2006, 2006; Udovicki et al., 2018). Although industrial countries are mostly located in the moderate continental climate belt and malnutrition is rare there, toxigenic *Aspergillus* species are moving constantly north due to climate change (see Figure 1; Battilani et al., 2016; Udovicki et al., 2018). Even nowadays, mycotoxin contaminations and mycotoxicoses are taken mainly as the problem of the Third World (Figure 1). Africa, South America, and other tropical countries have already been combating the ever-growing threat of mycotoxins for a long time. Even there, the types and the amounts of mycotoxins in the feed and food will be altered with the changing climate. To make things even worse, non-prioritized toxins can also emerge as new risks with unforeseeable effects and interactions. Unfortunately, big nations, organizations, or countries, like the World Health Organization (WHO), United States, China, or the European Union (EU) have different limiting values for mycotoxins (EC 1881/2006, 2006; Marasas, 1995), which makes any concerted actions by them quite difficult. During the last few years, several economical, health, and agricultural studies opened the question: what kind of pre- and post- harvest conditions and prevention methods would be manageable and safe for human and animal health (Shephard, 2008; Hamid et al., 2013; Mitchell et al., 2016)? As humans are on the top of the food chain, accumulation of mycotoxins clearly depends on animal consumption as well, so feed contamination should also be taken into account and thoroughly controlled. Nowadays, the globalization of food production systems can easily lead to accidental exposures of the consumers to multiple mycotoxins because (i) various mold infestations can affect the same crop concomitantly, (ii) additional infestations can occur during food processing, and (iii) customers can buy and consume contaminated foodstuffs bearing different mycotoxin contaminants. Importantly, all the above events can be separated both spatially and temporally. These palpable tendencies should raise the need for complex analytical and interdisciplinary studies in the future, especially when the changing climate represents a new global challenge to the food production and food safety regulatory systems (Gruber-Dorninger et al., 2019).

## Food Toxicology and Molecular Mechanism of Mycotoxins

Food toxicology is the field of science which deals with the toxicological effects of food components (Hussein and Brasel, 2001). Not surprisingly, food and feed also contain the most complex mixture of low-molecular-weight xenobiotics to which humans and animals are exposed. Because of the growing amount of evidence on the presence of mycotoxins in the feed and food chain, food toxicology should be considered seriously as an important discipline in combating mycotoxicoses (Shaw, 2014; Dellafiora et al., 2018).

The dose – response relationship specifies the magnitude of the response of an organism to exposure to a given chemical stimulus after a certain exposure time. Acute mycotoxicoses could be described with a rapid onset and a general response (Marroquín-Cardona et al., 2014). The relationship between the concentration of mycotoxins in food and the concentration of toxicologically active substances at the site of action could be characterized by toxicokinetics. The relationship between the concentration of toxicants at the site of action and the toxic effect at the level of molecules, tissues, or organs is determined by toxicodynamics (Dellafiora et al., 2018). All *Aspergillus* species can produce a wide range of mycotoxins, although each species has one predominant, characteristic toxin in many cases (Sweeney and Dobson, 1998). Because of the multivariate nature of mycotoxins and their co-occurrence in food and feed, co-ingested mycotoxins give rise usually to mixed symptoms coming from additive and synergistic effects (Marroquín-Cardona et al., 2014; Flores and González-Peñas, 2016; Dellafiora and Dall’Asta, 2017; Dellafiora et al., 2018). Brief toxicological aspects of *Aspergillus*-derived mycotoxins are described in the following.

### Aflatoxins

**FIGURE 2:**
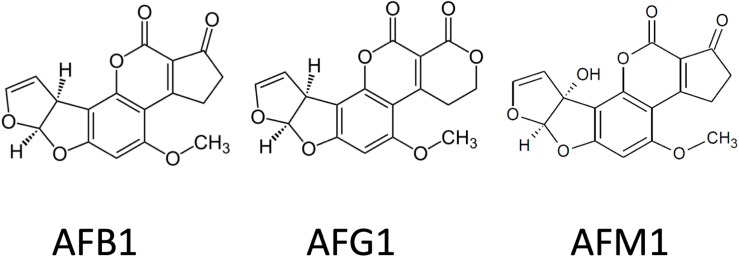
Chemical structure of some carcinogenic aflatoxins.

More than 20 types of aflatoxins (AFs) and their derivatives occur in nature, but mainly four, B1, B2, G1, and G2, are proved to be dangerous for humans and livestock (Wu et al., 2013; Smith et al., 2016; Udovicki et al., 2018; Rushing and Selim, 2019). AFs are furanocoumarins and are produced by various strains of *Aspergillus*, including *Aspergillus flavus*, *Aspergillus parasiticus*, *Aspergillus nomius*, and *Aspergillus pseudotamarii* as main AF producers (Figure 2; Council for Agricultural Science and Technology, 2003). Immunotoxic, carcinogenic, and mutagenic effects are mainly attributed to the presence of the lactone ring and the difuran ring (Vanhoutte et al., 2016). Aflatoxin B1 (AFB1) is the most carcinogenic and best-studied AF. Aflatoxin M1 (AFM1) is the 4-hydroxy derivative of AFB1, formed in the liver and excreted into the milk by the mammary glands of both humans and lactating animals that have been fed with AFB1-contaminated diet (Benkerroum, 2016; Cherkani-Hassani et al., 2016; Alshannaq et al., 2017). As it is also excreted in the urine, it is used frequently as a biomarker after AF exposure. AFB1 is metabolized in the liver by the cytochrome P450 enzyme system (CYPs) and a potent carcinogen derivative is aflatoxin B1-8,9-epoxide (AFB0), which has an *exo* and an *endo* isomer (Rushing and Selim, 2019). Primarily, the CYP3A4 and CYP1A2 are responsible for AF biotransformation, and mainly the *exo* isomer is formed, which has a highly electrophilic nature, perfect for spontaneous reactions with biological amines in nucleic acids and proteins (Rushing and Selim, 2019). In the case of DNA, AFB0 binds covalently to the N_7_ position on guanine, forming AFB1-N_7_-guanine adduct. The *endo* isomer has lower affinity than the *exo*, so AFB1-*exo*-8,9-epoxide is thought to be the major carcinogenic metabolite. Aflatoxicol (AFL) is the only metabolite that could go through the placenta and which is formed by the placenta itself. AFL is often found in the cytosolic fraction of liver preparations and thought to be a reservoir for AFB1, because it could be enzymatically converted back into AFB1, using the cytosolic NADPH system. That mechanism could be responsible for the AF-caused growth impairment, observed mainly in developing countries (Rushing and Selim, 2019).

Acute aflatoxicosis results in death while chronic exposure results in cancer, immunosuppression, and slowly manifesting pathological conditions (Phillips et al., 2002; Dharumadurai et al., 2011; Figure 4). Chronic aflatoxin poisoning leads to impaired DNA duplication in the bone marrow, which causes low leukocyte levels (Corrier, 1991; Fink-Gremmels, 1999; Benedict et al., 2016), which in turn gives rise to immunodeficiency and various infections. AFs also have a non-specific, cell multiplication inhibiting effect on other cell types (Bennett and Klich, 2003; Khlangwiset et al., 2011). This effect is the most prominent in the gastrointestinal tract, where an intact cell cycle is essential for the proper function of the digestive system (Liew and Mohd-Redzwan, 2018). The lethal dose (LD_50_) values for AFs are within the range of 0.5–10 mg/kg, depending on the chemistry of the derivative (Hymery et al., 2014). The primarily affected organ is the liver, and patients suffer from bile duct proliferation, centrilobular necrosis, hepatic lesions, and fatty acid infiltration, which often ends in liver cancer (Wu and Santella, 2012; Hymery et al., 2014; Saha Turna and Wu, 2019).

The International Agency for Research on Cancer (IARC) has classified aflatoxins, including AFB1, AFB2, AFG1, AFG2, and AFM1 as carcinogenic to humans, i.e., as GROUP 1 carcinogens (International Agency for Research on Cancer, 2012; Ostry et al., 2017). AFs and the metabolites produced by the hepatic CYP enzymes showed an interference with nucleotide pairing, which can lead to different genetic changes, large-scale chromosomal aberrations, or even to DNA strand breaks (Wild and Gong, 2009). The G→T transversion in codon 249 of the p53 gene causing an Arg249Ser mutation on p53 protein is one of the most common mutations found in human hepatocytes exposed to AFB1. Arg249Ser mutation enhances cell growth and clonal expansion and inhibits wild-type p53 activity and apoptosis (Dumenco et al., 1995; Forrester et al., 1995; Rushing and Selim, 2019). Glutathione conjugation catalyzed by GST (glutathione-S-transferase) of AFBO is a major route of detoxification, forming an inert metabolite that is not able to react with the DNA (Rushing and Selim, 2019). That conjugate is then converted into a mercapturic acid adduct *in vivo* and is then excreted in the urine (Moss et al., 1985; Rushing and Selim, 2019). Glutathione-*S*-transferase expression is higher in mouse than in other animals, which could be a reason why these rodents are more resistant to AFB1 exposure.

Aflatoxins can also damage the hepatocytes directly or through changing the expression of lipid metabolism connected genes (*Cpt1a*, *Lipc*, *Lcat*, *Scarb1*, and *Ahr*). The elevated cholesterol, triglyceride, and lipoprotein production can cause the deterioration of hepatocytes because of the increased metabolic need and anaerobic cell metabolism (Rotimi et al., 2017). The elevated lipid fraction and the changed HDL–LDL ratio in the blood can increase the possibility of coronary heart diseases. The death of hepatocytes will lead to acute hepatitis, which can cause liver failure and death or lower the chance of survival (Hamid et al., 2013). Patients with hepatitis have an impaired metabolism, which can result in malnutrition (Nurul Adilah et al., 2018). The lack of nutrients also leads to the depletion of reducing agents like glutathione and thus to the overall reduction of antioxidative capacity in hepatocytes. In the absence of nutrients, the hepatic tissue repair and regeneration cannot function properly and the liver failure is almost inevitable (Magnussen and Parsi, 2013).

### Ochratoxins

Ochratoxin A (OTA) was first described in 1965, and it is one of the most important mycotoxins (Heussner et al., 2015), which is produced mainly by *Aspergillus ochraceus*, *Aspergillus carbonarius* and *Aspergillus niger* as well as by *Penicillium verrucosum* (Ostry et al., 2013; Bui-Klimke and Wu, 2015). OTA is a pentaketide compound derived from a dihydrocoumarin family derivative coupled to β-phenylalanine (Zhu et al., 2017). IARC has classified OTA as a Group 2B carcinogen, which means that it is possibly carcinogenic to humans. OTA has also been reported as nephrotoxic, hepatotoxic, embryotoxic, teratogenic, neurotoxic, immunotoxic, and genotoxic (Pfohl-Leszkowicz and Manderville, 2007; Bui-Klimke and Wu, 2015). The symptoms of OTA poisoning are dose-dependent, and its carcinogenic properties are already well known in a variety of animal species.

The human aspects of OTA poisoning are not yet fully understood, although OTA in humans can cause kidney damage, cancer, or kidney failure, according to previous studies (Figure 4; Heussner et al., 2015). A well-reported case was the so called Balkan Endemic Nephropathy (BEN) (Barnes et al., 1977). Several various human nephropathies reported in countries as Bulgaria, Romania, Serbia, Croatia, Bosnia, Herzegovina, Slovenia, Macedonia, and Montenegro could be related to OTA (Reddy et al., 2010). African countries such as Congo, South Africa, Tunisia, Morocco, and Egypt struggled with similar cases. These effects of OTA were, however, not conclusive under laboratory conditions. Both the monitoring of OTA and the diagnosis of OTA-induced mycotoxicosis in humans rely on blood and urinary OTA levels. The BEN cases could not be related to the genetic background of the patients but, instead, to environmental factors like the mold-contaminated local grain (Pfohl-Leszkowicz and Manderville, 2007; Bui-Klimke and Wu, 2015). Surprisingly, chronic exposures to low OTA doses could even be more harmful than acute high-dose exposures (Pfohl-Leszkowicz and Manderville, 2007; Reddy et al., 2010). The most frequent way of OTA exposure is dietary intake (Reddy et al., 2010). Naturally and after biotransformation in the human body, more than 20 OTA derivatives exist. Importantly, OTA forms covalent DNA adducts through radical and benzoquinone intermediates. In addition, the OTA hydroquinone (OTHQ) metabolite can undergo an autoxidative process to generate the quinone electrophile OTA quinone (OTQ) that also reacts with DNA. Furthermore, the formation of OTQ or phenoxy and aryl radicals can result in increased reactive oxygen species (ROS) production that is responsible for its cytotoxicity. The mechanisms leading to OTA nephrotoxicity as well as its hepatotoxicity and immunotoxicity can be linked to the inhibition of protein synthesis, lipid peroxidation, and the modulation of the MAP kinase cascade, in a way similar to the exposure to pentachlorophenol derivatives (Heussner et al., 2015; Malir et al., 2016; Zhu et al., 2017).

### Emerging and Other Mycotoxins

Beside the toxins discussed above, *Aspergillus* species can also produce other toxic compounds that are not in the focus of food toxicology yet. They are, nonetheless, important and form an emerging branch of mycotoxicology and are already the objects of complex medical research projects in many cases.

#### Gliotoxin

**FIGURE 3:**
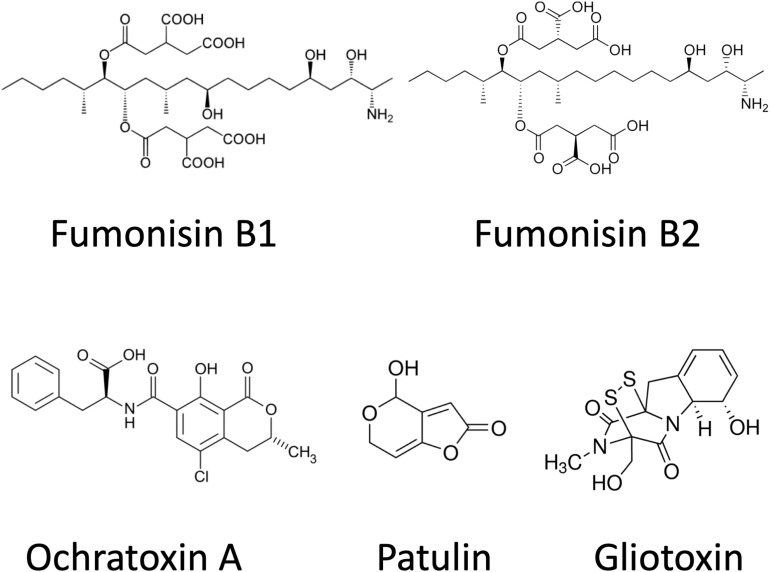
Chemical structures of ochratoxin A, patulin, gliotoxin, and fumonisins.

Gliotoxin (GTX) is often referred to as a virulence factor. It is produced mainly by *Aspergillus fumigatus*, although *A. terreus*, *A. flavus*, and *Aspergillus niger* are also able to synthesize it (Kwon-Chung and Sugui, 2008). GTX is a dipeptide and has a disulfide bridge across the piperazine ring, being a member of epipolythiodioxopiperazines (ETPs; Figure 3; Trown and Bilello, 1972). This molecular feature could function in cross-linking with cysteine residues in proteins, which results in the generation of ROS through redox cycling reactions. The outcome of these deleterious molecular processes is immunosuppression and necrosis. GTX also alters the tight junction structures by an unknown molecular mechanism and has a cytotoxic effect on astrocytes (Patel et al., 2018).

**FIGURE 4:**
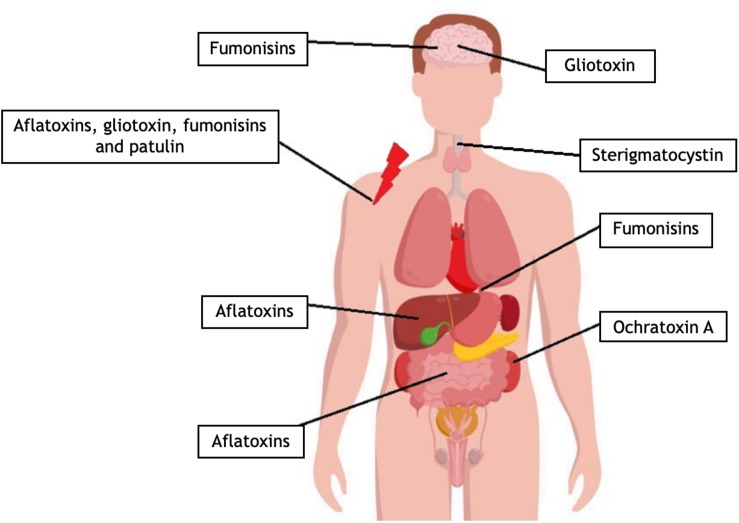
Toxicological effects of mycotoxins in the human body. Fumonisins can alter the sphingolipid metabolism, and it has an effect on the membrane of different cells like neurons. Fumonisins may increase the possibility of esophageal cancer formation. With different molecular pathways, aflatoxin, gliotoxin, fumonisin, and patulin can suppress several immunological mechanisms. Aflatoxins affect the pairing of nucleotides. Mutations of proto-oncogenes or tumor suppressor genes can cause liver cancer. Aflatoxin metabolites produced by the hepatic CYP enzymes can lead to chromosomal DNA strand breaks. Aflatoxins can inhibit cell proliferation. In the gut, mycotoxins can interfere with the regeneration of the gastrointestinal tract forming cells. Gliotoxin can penetrate the blood–brain barrier, and due to its cytotoxicity, it can damage the astrocytes. Sterigmatocystin may cause esophageal cancer. Ochratoxin A is nephrotoxic and can cause kidney damage, cancer, or renal failure. OTA was recently connected to Balkan Endemic Nephropathy (BEN) kidney disease and chronic interstitial nephropathy (for references, see the text).

Gliotoxin, like AFs, has an immunosuppressive effect, but the molecular mechanism is different. GTX in lower concentrations can inhibit the activation of inflammatory cells, the signaling and communication pathways between the leukocytes, the phagocytosis of macrophages, or the oxidative agent production of neutrophils and macrophages (Figure 4; Corrier, 1991). In higher concentrations (>250 ng/ml) GTX can induce apoptosis in leukocytes (Lewis et al., 2005). The GTX-producing human pathogenic fungi like *A. fumigatus* can evade the immunological responses. Other immunodeficiencies, as AIDS, chronic steroid treatment, alcohol abuse, and malnutrition can also be enhanced by GTX poisoning.

#### Fumonisins

Fumonisins are a group of related polyketide-derived, non-fluorescent mycotoxins. More than 53 different fumonisins have been reported so far (Marasas, 1995; Månsson et al., 2010; Nair, 2017). They can be divided into four main series (A, B, C, and P) but research has focused on the B series, mainly FB1, FB2, and FB3, which are the most abundant in nature (Mogensen et al., 2009). Fumonisin B compounds consist of a long hydroxylated hydrocarbon chain, which are decorated by tricarballylic acid and amino and methyl groups. FB2, FB3, and FB1 have different hydroxylation patterns (Kouzi et al., 2018). Fumonisins are structurally similar to cellular sphingolipids and, not surprisingly, they have been shown to inhibit sphingolipid biosynthesis at ceramide synthase (Marasas, 1995). The primary amino and tricarballylic acid groups of the toxin are responsible for the reaction with ceramide synthase. Fumonisin-induced toxicity often results in apoptosis, alteration in cytokine expression, or generation of oxidative stress (Kouzi et al., 2018). IARC has been classified FB1 in toxicity Group 2B as probably carcinogenic for people. *Aspergillus* species belonging to *Aspergillus* section *Nigri* are widely occurring species, and one of them, *A. niger*, is a highly important industrial organism in citric acid production. Black *Aspergilli* including *A. niger* and *A. welwitschiae* can be responsible for the FB2 (and FB4) contents observable in some foods and feeds as grapes, raisins, wine (Mogensen et al., 2009, 2010), onions (Varga et al., 2012; Gherbawy et al., 2015), and maize (Logrieco et al., 2014). However, *Fusarium verticillioides*, *Fusarium proliferatum*, and other *Fusarium* spp. cause higher fumonisin contaminations with FB1 (Frisvad et al., 2007; Kamle et al., 2019). Co-occurrence of fumonisin producing *Fusaria* and black *Aspergilli* in the kernels of maize may influence the observed FB1/FB2 ratios (Logrieco et al., 2011; Susca et al., 2014). Studies indicate that the fumonisins could be responsible for esophageal cancer in South Africa and have been shown to cause leukoencephalomalacia in horses and pulmonary edema in pigs (Kouzi et al., 2018). Fumonisins are also responsible for other diseases including neural tube defects, leukoencephalomalacia, pulmonary edema, hepatotoxicity, nephrotoxicity, or renal carcinogenesis (Nair, 2017; Figure 4). As sphingolipids are vital in regulating various cellular processes and they are a large family of metabolically linked signaling molecules, the acute and chronic toxicities of fumonisins are the result of the disruption of the sphingolipid metabolism and, as a result, the affected organs are very diverse. Recent findings also showed increased ROS production after fumonisin exposure, which may result in DNA damage and other enzyme defects but more research is needed to clarify the molecular backgrounds of these effects (Kouzi et al., 2018).

#### Sterigmatocystin

More than 50 fungal species can produce sterigmatocystin (STC), which, similar to AFs, is a polyketide mycotoxin. *A. flavus*, *A. parasiticus*, and *Aspergillus* section *Nidulantes*, subclade *Versicolores* are the most common source. Biosynthetic pathways of AFs and STC share many biosynthetic enzymes (Díaz Nieto et al., 2018). Since *A. nidulans* and *A. versicolor* are apparently unable to biotransform STC into O-methylsterigmatocystin, the direct precursor of AFB1 and AFG1, substrates colonized by these fungi can contain high amounts of STC. On the other hand, substrates invaded by *A. flavus* and *A. parasiticus* contain only low amounts of STC as most of it is converted into AFs (EFSA, 2013). According to different animal models and cell culture experiments, STC can also induce tumors; therefore, IARC classifies it in the Group 2B as possible human carcinogen (EFSA, 2013). In spite of this classification, the maximum acceptable levels of STC in food are not regulated worldwide. The acute oral toxicity of STC is relatively low, with LD_50_ values varying between 120 and 166 mg/kg bw. After oral exposure, premalignant and malignant lesions, such as hepatocellular carcinomas and angiosarcomas in the brown fat, have been reported. STC is genotoxic and carcinogenic, although the carcinogenic potency of STC is approximately three orders of magnitude lower than that of AFB1. STC is metabolized in the liver and lung by various CYP enzymes into different hydroxy metabolites (Díaz Nieto et al., 2018), and STC-metabolites are excreted in the bile and the urine (EFSA, 2013). The mutagenicity of STC is due to these reactive epoxi-adducts, which can covalently bind to DNA and generate the STC-N_7_-guanine adducts. Another mechanism was also proposed by Pfeiffer et al. (2014), who suggested that the hydroxylation of the aromatic ring generates a catechol, which could react with DNA. This was based on the finding that in liver microsomes of humans and rats the catechol was mainly formed while the epoxide was formed in smaller amounts. Intensive research has been launched recently on the role of STC in human esophageal and gastric cancers (Figure 4). *In vivo* experiments were performed in a rat model system, and these findings confirmed the conclusions previously drawn from experiments on human-derived cell lines (Tong et al., 2013; Díaz Nieto et al., 2018). It has been demonstrated in a human immortalized bronchial epithelial cell line that STC could induce DNA double-strand breaks, which may lead to adenocarcinomas.

#### Patulin

Patulin (PAT) is produced by many different molds, predominantly by *Penicillium* spp. (Puel et al., 2010; Frisvad, 2018; Vidal et al., 2019) but, occasionally, by some *Byssochlamys* (Sant’Ana et al., 2010; Frisvad, 2018) and *Aspergillus* spp., including *A. giganteus*, *A. longivesica*, and *A. clavatus* (Varga et al., 2007; Pal et al., 2017; Frisvad, 2018) as well. Chemically, PAT is a water-soluble, colorless, polyketide lactone (Figure 3), which is thought to exert its toxicity through reacting with thiol groups (cysteine, glutathione, thiol moieties of proteins) in the cytoplasm (Pal et al., 2017). In addition to its antibacterial, antiviral, and antiprotozoal activities, PAT was also reclassified as a mycotoxin.

Because PAT also possesses acute toxicity, teratogenicity, and mutagenicity properties at the same time (Puel et al., 2010), the emerging symptoms of PAT mycotoxicoses are typically non-specific but mostly connected to the enzyme inhibitions (Pal et al., 2017). The affected enzymes usually take part in digestion, metabolism, and energy production. Intestinal disorders, decreased food intake, decreased weight, together with altered lipid metabolism could be observed in many animal models. PAT can also compromise the immune system and modify the different response mechanisms of the host (Corrier, 1991), and also inhibits transcription, translation, and DNA synthesis in leukocytes (Mahfoud et al., 2002; Figure 4). *In vitro* studies have demonstrated that PAT inhibits macrophage functions like reduced rate of protein synthesis of lysosomal enzymes and cytokines, altered membrane functions, and significantly decreased ROS production, defects in phagosome–lysosome fusion, and phagocytosis (Wichmann et al., 2002).

## Occurrence of *Aspergillus*-Derived Mycotoxins in the Feed and Food Chain

**FIGURE 5:**
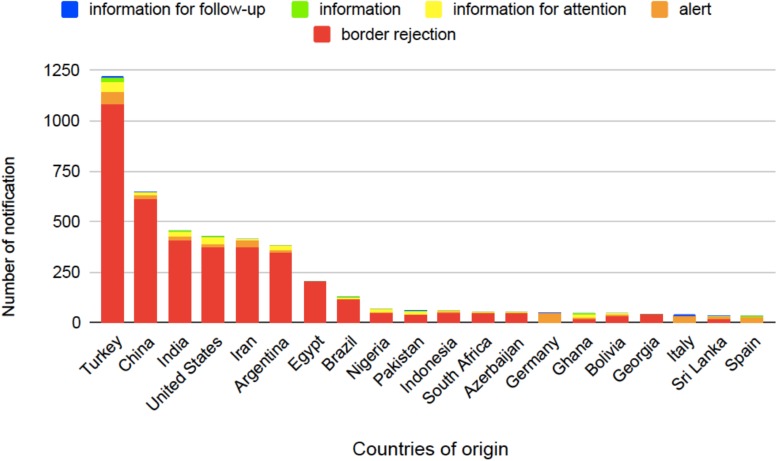
Countries of origin for aflatoxin-related notifications in food based on the European Union (EU) Rapid Alert System for Food and Feed (RASFF) database from 1st January 2009 until 27th June 2019 (European Parliament and of the Council, 2002).

Several studies have been carried out in order to set appropriate food safety regulations and recommendations (see Table 1). These regulatory actions, however, must pursue reasonable trade-offs to avoid unreasonable food wasting and to regulate trade economic effects (Marroquín-Cardona et al., 2014; Dellafiora et al., 2018). About 20–25% of the harvested fruits and vegetables are lost due to various post-harvest diseases primarily caused by molds even in developed countries, and this loss can even be more severe in developing countries (Medeiros et al., 2012). The average annual economic loss attributable to mycotoxin contamination is about 1 billion USD in the United States alone (Amaike and Keller, 2011). AFs are leading the list of the most harmful mycotoxins when economic losses as well as agricultural and health threats are considered and evaluated (Amaike and Keller, 2011). The European Union (EU) Rapid Alert System for Food and Feed (RASFF) was created in 1979, which is currently based on the Regulation 178/2002 (European Parliament and of the Council, 2002). The EU members can exchange information on hazards in food through the Alarm System. Six types of notifications are in use: alerts, information, information for attention, information for follow-up, border rejections, and news; however, the last one is not available for AFs. When a toxin-containing food appears on the market, rapid action, like product recall, is necessary and an alert notification is sent to RASFF as well. Nearly 90% of the reported risks come from outside of the EU (Figure 5); thus, border rejections are sent to all external border posts of the EU to secure that the contaminated product does not enter through other entry points (Pigłowski, 2018).

**TABLE 1:** *Aspergillus*-derived mycotoxins and *Aspergillus* spp. that produce them, high-risk foods, maximum levels in EU, FDA levels, and guidance values by WHO.

**Mycotoxin**	**Producing fungi**	**High-risk foods**	**EU Maximum Level**	**FDA levels**	**Guidance value by WHO**
Aflatoxins (AFB1, AFB2) (AFG1, AFG2, AFM1)	*A. flavus, A. parasiticus A. parasiticus*	Maize, wheat, barley and other cereals, peanuts and oil seeds, cottonseed, coffee and cocoa beans, figs and dried fruits, spices, milk and dairy products	AFB1 2–8 μg/kg sum of AFs 4 15 μg/kg AFM1 0.025–0.050 μg/kg baby and infant foods 0.10 μg/kg	Foods 20 μg/kg Milk 0.5 μg/L	PTWI is not established
Fumonisins (FB2, FB4)	Predominantly *Fusarium-*derived mycotoxins, but also produced by *A. welwitschiae* and *A. niger*	Maize, wheat, barley, rice, millet, oats, coffee beans, grapes	Unprocessed maize 4 mg/kg maize-based foods 1 mg/kg cereals and snacks 800 μg/kg baby and infant foods 200 μg/kg	2–4 mg/kg	PMTDI 2 μg/kg bw
Ochratoxin A (OTA)	*A. ochraceus, A. carbonarius, A. niger*	Maize, wheat, barley, legumes, oil seeds, peanuts, coffee beans, cocoa beans, dried fruits, grape juice and wine, spices, meat products	Unprocessed cereals 3 μg/kg coffee beans 5 μg/kg dried fruit 10 μg/kg juice and wine 2 μg/L dried spices 15 μg/kg baby and dietary foods 0.5 μg/kg	No level set (Mitchell et al., 2016)	PTWI 112 ng/kg bw
Patulin (PAT)	Predominantly *Penicillium-*derived mycotoxin, occasionally also produced by *A. clavatus* infestation of feed and food stuffs	Apples, grapes, many fruits, juice, cider, tomatoes	Fruit juice and cider 50 μg/L baby foods 0.10 μg/kg	Apple juice 50 μg/L	PMTDI 0.4 μg/kg bw
Sterigmatocystin (STC)	*A. versicolor, A. nidulans*	Maize, wheat, peanuts, oil seeds, coffee beans, milk and dairy products	No data	No data	PTWI is not established
Gliotoxin (GTX)	*A. fumigatus*	Cattle feed, mussel	No data	No data	PTWI is not established

*PMTDI, provisional maximum tolerable daily intake; PTWI, provisional tolerable weekly intake. European Union (Regulation 1881/2006), US FDA–chemical contaminants, metals, natural toxins, and pesticides guidance documents and regulations, JECFA–Joint FAO/WHO expert committee on food additives.*

**FIGURE 6:**
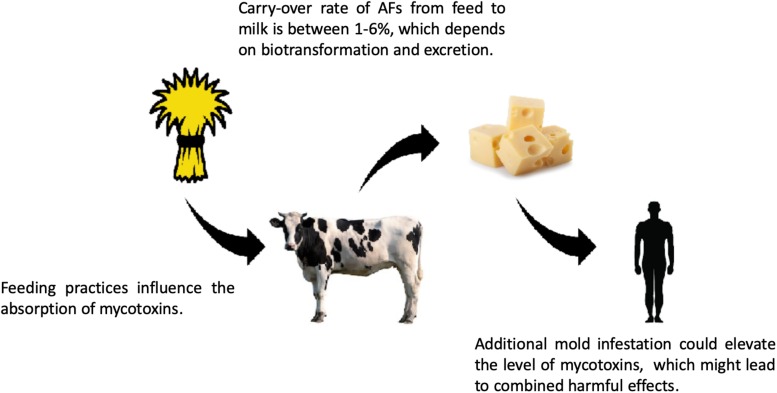
Risk of mycotoxin exposure in the feed and food chain. Mycotoxins, like aflatoxins (e.g., AFB1), go through biotransformation in the livestock and different metabolites are produced, such as AFM1, which can be excreted into the milk, where AFM1 can bind to casein. After digestion, AFM1 is released from the casein – AFM1 complexes. The consumption of high amounts of dairy products contaminated with AFM1 can lead to acute mycotoxicosis (for references, see the text). The carry-over rates of mycotoxins show seasonal differences, and there are other diverse factors influencing the prevalence of carry-over, e.g., the quantity of mycotoxins in the feed and the excreted amount of toxin in the milk. The geographical location and feeding practice could also affect the carry-over rates, which could be even 6%, regarding AFs (Völkel et al., 2011).

January 2009 the most commonly infected plants are cereal crops, like maize and wheat, as well as cotton, soybean, and different forms of nuts, especially groundnuts (Jelinek et al., 1989; Dharumadurai et al., 2011). Fungal growth and toxin contamination are the consequence of interactions among fungi, the host, and the environment. As mentioned above, animals can act as transmitting agents, as meat, milk, or eggs can pass AFs to species in the food chain (Völkel et al., 2011; Figure 6). Food processing can increase or decrease the concentration of AFs. For instance, AFM1 is associated with protein fractions of the milk. It is worth noting that AFM1 is heat-stable and binds to casein and, hence, tends to accumulate in cheese (Sengun et al., 2008; Busman et al., 2015; Benkerroum, 2016). Milk products like different types of cheese can have three to five times higher concentration compared to bulk milk, while butter or yogurt processing can significantly decrease the concentration (Govaris et al., 2001; Iha et al., 2013). Another group reported that cocoa butter transmitted no infection from the originally infected cocoa beans (Turcotte et al., 2013). Tropical and Mediterranean climates facilitate the production of AFs, as toxin production of *A. flavus* and *A. parasiticus* is reported between 28 and 35°C (average, 30°C), but some fungi stop the synthesis of AFs above 36°C (Table 2; Yu et al., 2008). These factors mean that ingredients from these regions have higher risk of AFs contamination (Battilani et al., 2016).

**TABLE 2:** Growth conditions of some *Aspergillus* species and their optimum temperature for mycotoxin production.

**Fungi**	**Mycotoxins**	**Growth temperature**	**Optimal toxin production temperature**	**Optimal growth pH**	**Water activity**	**References**
*A. flavus*	AFB1, AFB2	25–30°C	28–35°C	5–6	0.94–0.97	Lahouar et al., 2016; Stein and Bulboacă, 2017; Frisvad et al., 2019
*A. parasiticus*	AFB1, AFB2, AFG1, AFG2	15–33°C	28–35°C	5	0.95–0.99	Mannaa and Kim, 2017; Stein and Bulboacă, 2017; Frisvad et al., 2019
*A. niger*	FB2	24–37°C	25–30°C	5	0.97–0.99	Mogensen et al., 2009; Passamani et al., 2014
*A. versicolor*	STC	30°C	23–29°C	3.1–10.2	Min. 0.76	Veršilovskis and De Saeger, 2010; Stein and Bulboacă, 2017
*A. ochraceus*	OTA	24–37°C	31°C	3–10	Min. 0.8	Reddy et al., 2010
*A. clavatus*	PAT	24–26°C	25°C	4.7	0.87	Zutz et al., 2013
*A. fumigatus*	GTX	under 42°C	37°C	7.35–7.45	0.92–0.97	Alonso et al., 2016

*These representative data could be influenced by different environmental circumstances.*

Not surprisingly, data for dietary intake of mycotoxins are available in many countries for different age cohorts including children and infants (Marin et al., 2013). The physiological effects of mycotoxins and the assessed health risks for children and infants are different from those of adults (Sherif et al., 2009; Raiola et al., 2015). A recent study on Gambian infants revealed an effect of AF exposure on the growth of infants (Watson et al., 2018). Although further research is needed, AF content of baby food might cause growth impairment in children. Even though the WHO designated AFB1 and AFM1 as Class 1 carcinogens, some levels of consumption can be tolerated. The safe content of the derivatives of AFs depends on the foodstuffs. Limits in the EU are between 2 and 8 μg/kg AFB1 in foodstuffs dedicated for adults and 0.1 μg/kg AFB1 in baby foods for infants and toddlers. Regarding AFM1, the limits are lower, particularly 0.025 μg/kg in dairy products, including infant formula. The overall content of AFB1, AFB2, AFG1, and AFG2 in different foodstuffs is not allowed to be higher than 15 μg/kg (EC 1881/2006, 2006). Risk assessment analysis indicated that the hazard index for children under the age of 3 years was considerably higher than that for adults, which supports the need for more effective mycotoxin risk assessment and self-control strategies in the milk industry (Farkas et al., 2014; Trevisani et al., 2014; Kerekes et al., 2016; Ortiz et al., 2018).

The ochratoxins produced by strains of *A. ochraceus*, *A. carbonarius*, and *A. niger* are often present together in food. OTA can be found in a variety of agricultural products, especially in cereals, grapes, and related products (Streit et al., 2012; Tsitsigiannis et al., 2012). This mycotoxin occurs naturally and is widespread around the world, but mainly in the Mediterranean Basin, including Italy, Spain, and Greece (Covarelli et al., 2012; Somma et al., 2012; Perrone et al., 2013; Arroyo-Manzanares et al., 2019), and furthermore, in several African countries like Cameroon, Senegal, Benin, and Nigeria (Rodrigues et al., 2011; Tang et al., 2019). OTA can also be considered as a potentially emerging mycotoxin in Central Europe due to the climate change (Tóth et al., 2013; Pleadin et al., 2017; Udovicki et al., 2018). The most common types of food bearing OTA are cereal grains, oil seeds and tree nuts, wine, wine grapes and dried fruits, spices, herbs and herbal teas, cocoa powder, and coffee beans. Ochratoxins are food-borne mycotoxins, and this post-harvest contamination can appear if crop-drying practices are suboptimal and delayed (Reddy et al., 2010; Bui-Klimke and Wu, 2015). Analysis of several food and feed samples were performed with enzyme immunoassays, which gave detection limits of 0.5 to 5 μg/kg. Intoxicated dry beans could bear 5–30 μg/kg, whereas maize can bear 10–50 μg/kg, and green coffee beans contain 18–48 μg/kg. Even as low as 0.16 μg/L and 0.24 μg/L of OTA could be detected in South African white and red wines, where the detection limit was above 0.01 μg/L (Reddy et al., 2010). Ordinary food processing is not able to eliminate or substantially reduce the quantity of OTA in foods and beverages. Furthermore, processed food products such as sausages and bread were also found to contain OTA since it is a chemically very stable compound. The EU set the maximum permissible levels of OTA in unprocessed cereals at 3 μg/kg, in roasted coffee beans at 5 μg/kg, in dried fruits at 10 μg/kg, in fruit juice and wine at 2 μg/kg, in dried spices at 15 μg/kg, and in dietary and baby foods at 0.5 μg/kg (EC 1881/2006, 2006). The United States Food and Drug Administration (FDA) has not set maximum regulatory limits for OTA in food (Mitchell et al., 2016). Based on a detailed WHO risk assessment, including hazard identification, hazard characterization, exposure assessment, and risk characterization, the Joint Expert Committee on Food Additives (JECFA) issued an official statement on OTA and have set provisional tolerable weekly intake at 112 ng/kg bw, which was later rounded down to 100 ng/kg bw. The limit was set based on various dose–response studies on animals. The average weekly OTA intake in Europe is 8–17 ng/kg bw, being well below the advised limit (Bui-Klimke and Wu, 2015).

Patulin is produced by many different molds, which need special, e.g., dirty, wet environments for spreading (Puel et al., 2010; Ioi et al., 2017). Although mostly *Penicillium* spp. have been isolated from food with PAT contamination in the moderate climate belt, some recent studies have provided us with new insights into PAT occurrence in food, which are mainly connected to climate change. Inadequately stored cereals, e.g., under high moisture conditions, can lead to the colonization by *A. clavatus*, which is also responsible for the PAT contents of food in tropical and sub-tropical regions. It is hard to estimate the contribution of these molds to the PAT contents of foods and feeds precisely but the role of *Aspergillus* spp. in global PAT exposures should not be underestimated. Furthermore, *A. clavatus* may also colonize malted barley and wheat, which might also contribute to the appearance of PAT in the feed and food chain (Lopez-Diaz and Flannigan, 1997; Loretti et al., 2003; Sabater-Vilar et al., 2004). Different food products, like vegetables, rotting apples, grains, and fruits may contain primarily *Penicillium*-derived PAT (Puel et al., 2010; Wright, 2015; Frisvad, 2018; Vidal et al., 2019). As this is a quite stable secondary metabolite, it can withstand various harsh processing steps, such as milling and heating. Apples and apple derivatives have the highest concentration of PAT, and a maximum of 16 mg/kg has been reported so far (Pal et al., 2017). Although the incidence of PAT contamination is fairly high worldwide (Schatzmayr and Streit, 2013) commercial apple juices normally contain less than 10 μg/kg of PAT (Pal et al., 2017). Because PAT remains stable during apple processing, PAT detection is often used as a quality control parameter, indicating whether or not moldy apple was processed (Karlovsky et al., 2016). During ethanol fermentation, *Saccharomyces cerevisiae* can destroy PAT and, hence, ciders and other fermented fruit drinks will not contain this toxin (Yu et al., 2008), except when fresh fruit juice is added to the cider after fermentation. Due to its toxicity and potential harm to human health, according to JECFA, the provisional maximum tolerable daily intake of PAT is 0.4 μg/kg bw. PAT contaminations present in different food products are mainly hazardous for special age cohorts, such as infants and elderly people and also for gravidae. Since 2006, the European Commission and China have set the maximum limit for PAT to 50 μg/kg in fruits, while for products dedicated to younger people, the limit has been set to 10 μg/kg (EC 1881/2006, 2006; Ji et al., 2017).

Fumonisins are among the most significant agricultural toxin. Although these mycotoxins are mainly produced by *Fusarium* species, like *F. verticillioides* and *F. proliferatum*, this paper focuses mostly on fumonisins produced by *Aspergillus* species (Kamle et al., 2019). Fumonisins can cause serious loss to agricultural production of cereals both in the field and during storage and can be dangerous to animals and humans as well (Mudili et al., 2014). It has been shown that *A. niger* can be responsible for the presence of FB2 and FB4 (Varga et al., 2010). Since grapes, wines, dried fruits, and grape-derived products have a significant importance worldwide, the presence of *A. niger* and *A. welwitschiae* in the global grape and wine production chain has a high importance. When the temperature is below 30°C, several molds are responsible for the observed varying mycotoxin exposures, but when the temperature is higher than 37°C, predominantly black *Aspergilli* are responsible for FB2 and FB4 contents of these foods and drinks. The spreading of these species is even faster when the storing conditions are not optimal, and physical damages on the berries also help fungal invasion (Logrieco et al., 2011; Storari et al., 2012; Onami et al., 2018). Other commonly infected food grains are maize, wheat, barley, rice, millet, oats, and rye, but fumonisins are present in coffee beans, too (Palencia et al., 2010; Varga et al., 2010; Mudili et al., 2014). The most endangered species are horses, pigs, and humans through direct ingestion. Importantly, Mediterranean climate supports the spread of FB2 producer black *Aspergilli*, as their optimum temperature for growth lies between 25 and 30°C, with the upper and lower limits of 42 and 12°C (Mogensen et al., 2009).

Fumonisins are recognized by authorities and official limit values have been issued. FDA has set the safe intake limit to 4000 μg/kg for food products containing whole maize grains and 2000 μg/kg for products made with dried milled maize products. Animal feed limits depend on the targeted animal, so the limits can range from 5 to 100 mg/kg (FDA, 2001). JECFA and the European Commission Scientific Committee for Food have set the tolerable daily intake level of FB1, FB2, FB3, or their combination at 2 μg/kg bw. The EU has defined the maximum permissible levels for the sum of FB1+FB2 in unprocessed maize at 4000 μg/kg, in maize-based foods at 1000 μg/kg, and in cereals or snacks at 800 μg/kg. The maximum limit is 200 μg/kg in processed foods for infants and toddlers (Commission of the European Communities, 2006). Similar US regulations set 2000–4000 μg/kg levels for the sum of FB1+FB2+FB3 depending on the foodstuff (Bryła et al., 2017; Zhang et al., 2018).

Sterigmatocystin producing *Aspergillus* species, mostly *A. versicolor*, infect mainly grains and grain products. Part of the STC content in the food and feed are usually converted to AFs by aflatoxigenic species, e.g., *A. nidulans.* The impact of STC may appear smaller than AFs in the case of human intake, but the importance of STC cannot be excluded (EFSA, 2013). The occurrence of STC has been shown in cheese quite often because AF-producing fungi are rarely present there. Previous STC measurements in cheese found toxin levels from 5 to 600 μg/kg (Díaz Nieto et al., 2018). STC occurrence in spices (fennel sample, red pepper, black pepper, and caraway seeds) was also reported from African and Asian countries. For cereals, STC was reported in barley, wheat, rye, and oat, concentrations being around 10–60 μg/kg from some European countries. As traditional Chinese medicine is based on plants, STC was also reported in these medicinal plant products, too. We cannot state that STC occurrence in cheese is because of the feed as the rate of carry-over of STC into milk when ruminants are exposed to contaminated feed has not been inevitably proved. Moreover, no information is available about the transfer of STC and/or its metabolites into other animal products such as meat and eggs. The exact toxicity of STC in livestock is not clear, as no signs of toxicity were observed in sheep, when a feeding trial at the highest dose were performed (16 mg/kg STC in feed, estimated as equivalent to 0.3 mg/kg bw per day) (EFSA, 2013; Díaz Nieto et al., 2018). As risk characterization is not possible for STC, several international organizations recommend that more accurate data for STC in food and feed across European countries need to be collected. In case of food, methods with an LOQ (limit of quantification) of less than 1.5 μg/kg should be applied, whereas for feed, the available information is insufficient to make a recommendation. The development of suitable certified reference materials and/or proficiency tests to support analytical methodology should be encouraged (EFSA, 2013; Díaz Nieto et al., 2018). As the structure of STC and AFs are similar and metabolites are often common, analytical method development (immunoassays, isotope assays, etc.) and differentiation assays are needed to differentiate between these mycotoxins.

## Prevention Strategies of Mycotoxicoses

Current possibilities for the treatment of mycotoxin poisoning are still quite limited and are not specific. The best solution is, therefore, to prevent mycotoxins to enter the feed and food chain (Wagacha and Muthomi, 2008; Milićević et al., 2010; Kumar et al., 2017; Ortiz et al., 2018; Arroyo-Manzanares et al., 2019). The completely mycotoxin-free food and feed industry is, most likely, an irrational goal but the minimization of mold infestations and toxin deposition in the different agricultural products may be possible and can effectively prevent mycotoxin poisoning (Udomkun et al., 2017). It is important to state that the mold infestation is not equal to mycotoxin contamination. But the defense against all molds is favorable due to their effect on the economy (Wu and Khlangwiset, 2010; Ehrlich, 2014).

*Aspergillus* species can enter the food and feed chain at many stations of the industry (Gallo et al., 2015). The complex production systems, climate change, economic processes, and the resilience of the mycotoxins make it difficult to establish secure prevention protocols, sampling methods, and an international pipeline (Wild and Gong, 2009; Tasheva-Petkova et al., 2014; Bandyopadhyay et al., 2016; Paterson et al., 2017). The diverse factors that have an effect on the agricultural products can be divided into two groups: the pre-harvest and post-harvest circumstances (Jouany, 2007). Pre-harvesting factors include the production of crops, growing conditions, and the prevention of mold infestations in crops and other agricultural products (Kabak et al., 2006). Masked mycotoxins may mount an even greater risk to the consumers. It is well known that mold-infected plants may alter the chemical structures of mycotoxins as part of their defense mechanism against xenobiotics (Berthiller et al., 2013). The modified mycotoxins can generate deposits in the plant tissues and may remain hidden for conventional analytics. These masked mycotoxins might pose additional threats to human health and also represent further challenges to both global food safety and the scientific community working in this field. Obviously, to gain reliable and reproducible data on the masked mycotoxins present in feed and food, we need new analytical methods and also novel *in vivo* experiments. To lessen the possibility of mycotoxin exposures, it is important to raise awareness among the food- and feed-producing countries with educational campaigns. There are numerous options to lower the mycotoxin content of crops before harvest (Sundh and Goettel, 2013; Mahuku et al., 2019). Preventing mold infestations, limiting the spread of molds to other plants, or neutralizing the mycotoxins already at pre-harvest are all good examples and may hold great potentials. Competitive but atoxigenic mold species and variants can supersede toxin-producing *Aspergillus* species and, hence, are suitable candidates in the elaboration of various biocontrol strategies (Kagot et al., 2019). Large-scale monoculture farming is highly prone to mold infestations, and this tendency may strengthen further with changing climate. Cultivating more diverse crop variants with different harvest dates on smaller areas can effectively mitigate the risks of subsequent mold infestations. There are possibilities to reduce the mycotoxin production even if the mold infestation is present in crops (Pfliegler et al., 2015). Co-cultivating the crops with genetically modified plants or microorganisms might alter the chemical structure of mycotoxins *via* changed metabolic pathways as part of the defense against xenobiotics (Berthiller et al., 2013). Vitamin C may regulate the genes of mycotoxin production, inhibiting the expression of toxin-producing enzymes (Akbari Dana et al., 2018). With polyculture farming on timed planting and with modern methods like environmental stressors to prevent the infestation, the economical and medical effects of the mold contamination and mycotoxins could be minimized (Abramson et al., 1997; Atehnkeng et al., 2014). Before the time of harvest, an extensive examination of the crops should precede any other procedures (Cleveland et al., 2003), since after harvest it is much harder to reveal the contamination. The infected field should be decontaminated by immediate harvesting and discarding the contaminated crops to prevent further spreading.

The largest part of the threats is the post-harvest factors. These include the harvesting criteria, the transporting circumstances, the storage conditions before, and, after the processing steps, the sampling methods, the inspections and toxin detection protocols, and the international pipelines and regulations about the amount of the mycotoxins contained in the foodstuffs (Zain, 2011). The circumstances of storage are also crucial. Sorting before the storage of crops is essential (Fandohan et al., 2005) since in large storage facilities the mold infestation can spread more easily between the different portions of the harvested crops (Hell et al., 2000; Williams et al., 2014). On the other hand, the correct cleanliness of the storage buildings is also critical (Adda et al., 2011), since if the storage conditions are not correct or even favorable for the growing and spreading of mold, it could lead to huge economical and financial losses or even medical crises (Hussein and Brasel, 2001; Khlangwiset and Wu, 2010). Inappropriately chosen storage parameters like concomitantly high temperature and humidity can propagate mold infestations. Therefore, ingredients should be dried and/or cooled to prevent or at least limit fungal growth.

International pipelines and regulations have already been put into operation to find the occurring mold infestation and mycotoxin contamination as early as possible, but not everyone keeps the rules. In the current ecological situation, the ingredients of a product may come from all over the world. In the case of such multifactorial systems, it is even more difficult to control every aspect and, therefore, the ingredients should be investigated individually.

If the mold infestation remained undetected and the mycotoxin deposits are already formed, there are still possibilities to lower the toxin levels (Yang et al., 2014; Udomkun et al., 2017; Omotayo et al., 2019). Here, we outline the advantages and disadvantages of some mycotoxin decontaminating methods currently used in the agriculture and food industry and also aim to evaluate some foreseeable future tendencies in this field. Even though the toxins are heat-stable in a 150–200°C temperature range, their amount can still be lowered effectively by heating (Herzallah et al., 2008). This amount of heat can be problematic; in the case of heat-sensitive substances, the administered heat has therefore to be limited. Because of the remarkable heat stability of the *Aspergillus*-derived toxins and the high thermal sensitivity of some valuable nutrients and vitamins, any possibility for decontamination by heating should be considered with care. Under mild conditions, the efficiency of mycotoxin decomposition might be low because most mycotoxins are heat-resistant within the range of usual food-processing temperatures (80–121°C) (Bullerman and Bianchini, 2007; Kabak, 2009; Karlovsky et al., 2016).

Ionized radiation produced by gamma rays can also be used to lower the toxin levels (Ghanem et al., 2008; Jalili et al., 2010). As the large-scale application of this technique, it is quite difficult and it is usually applied as the last step in the food production, when the commodities have already been packed. In 2015, the International Atomic Energy Agency (IAEA) in partnership with the Food and Agriculture Organization of the United Nations (FAO) released the manual of good practice in food irradiation aiming at improving food irradiation practices worldwide, with a focus on developing countries (Di Stefano and Pitonzo, 2014). The dose for package sterilization is set between 10 and 20 kGy while the different foodstuffs like dried materials or spices are irradiated with 30–50 Gy. Any overdosing on gamma rays is contraindicated because it may induce the degradation of valuable nutrients and the formation of other toxic compounds. While this is a good method to lower the toxin content, in the case of rural food production when the crops are harvested for strictly personal use, it is not perfect. However, portable food-irradiation machines are accessible, although problems with financing and operating difficulties limits their usage (Roberts, 2016). Although irradiation tools may be quite complicated and may require a more advanced technical background, irradiation may represent a reliable and safe alternative for the decontamination of *Aspergillus*-derived mycotoxins in the future. There is also a more complex side of the reduction of mycotoxins by gamma irradiation. Especially in the case of high starting toxin concentration, radiolytic mycotoxin forms may be generated due to irradiation (Wang et al., 2011; Yang, 2019). Although the toxicological effects of the intact toxins and their radiolytic decomposition derivatives were compared, the radiolytes had significantly less impact on human health; the possible toxicological effects of the latter need further investigations. In the future, the foreseeable increases in the mycotoxin contents of different food commodities could bring the effects of these radiolytic mycotoxin degradation products into the spotlight.

Ozonation can also be an effective and reliable detoxification method. In the case of ozonation, oxygen radicals are generated through splitting of reactive ozone molecules, which then affect different contaminants. The application of ozone can be in both gas and liquid forms. One downside of this method is that the effective ranges of these radicals are short, and, hence, they cannot penetrate deeply into the different substances. The treatments must be used on a large surface, which is only achievable in the end of food production, just before the packaging. This protocol has the same disadvantage as irradiation does, as it cannot be applied on large quantities of foodstuff at the same time.

Besides physical toxin reductions, there are chemical substances available to change the properties of the mycotoxins and lower their physiological activities (Bryła et al., 2017). These methods are very popular due to the fact that most of the effective chemical components like citric, lactic, tartaric, hydrochloric, succinic, acetic, and formic acids are already in use in the food industry (Méndez-Albores et al., 2005, 2009; Wu and Khlangwiset, 2010). The chemical treatment can be acidification, ammonization, or ozonation (Karaca and Velioglu, 2014). Every procedure can be accelerated with increased temperature; otherwise, these methods would take days.

There are also biological methods to prevent and neutralize mycotoxins in food and feed stuffs (Komala et al., 2012; Quiles et al., 2015; Sultana et al., 2015). Biocontrol methods can give rise to the most effective prevention techniques in the future, and some methods have already been used with promising results. These protocols use different biocontrol agents (BCAs), which can modulate mycotoxin contaminations in various ways. These agents can be different microorganisms like other, atoxigenic but highly competitive, fungi, which can limit the spreading of the mycotoxin producer strains. One possibility is the inoculation of different microorganisms like *Lactobacillus* or *Saccharomyces* into the toxin-contaminated foodstuff (Tsitsigiannis et al., 2012). In addition, the application of yeasts in various technological processes may have a direct inhibitory effect on toxin production of certain molds, which is independent of their growth suppressing effect (Pfliegler et al., 2015). Other genetically modified BCAs can produce different substances like Vitamin C, which can silence the gene clusters responsible for mycotoxin productions. Furthermore, different enzymes obtained from various *Bacillus* species showed high efficiency, but they have not been tested on a large scale. Plant extracts with various enzymes might also be effective. The different methods work synergistically, through the degradation of the toxin, decreasing the active form of the mycotoxin or just binding to the toxin and reducing the free toxin ratio. These procedures are fairly effective, but their timescale is too long (48–72 h) and the methods are difficult to apply on large quantities, which mostly excludes them from industrial applications.

The combination of different methods can lead to reliable protocols that can be used to reduce the mycotoxin levels in the contaminated food and feed (Udomkun et al., 2017). The combinations can also be effective in cases when the properties of the target material limit the use of some toxin-decreasing procedures. The detoxification methods are essential in the fight against the mycotoxins but the wide array of toxin types and their different effects and physical and chemical properties make it difficult to find a universal solution (Omotayo et al., 2019). The best solution is to minimize the occurrence of mycotoxins in the food and feed industry.

## Medical Aspects of *Aspergillus*-Derived Mycotoxins

Despite prevention methods and strict regulations, mycotoxins are still present in the feed and food chain, and the diseases caused by dietary toxic fungal exposures are called mycotoxicoses (Peraica et al., 1999). The processes of mycotoxin poisonings have been partially cleared, but due to the multivariate nature of the food and feed contaminations and to their not yet fully understood metabolisms, the human side of poisoning needs further investigations. The medical data presented here are mainly acquired from large-scale toxin exposures as those recorded in the acute poisoning outbreak in Kenya in 2004 with 125 deaths (Probst et al., 2007), in Tanzania during 2016 with 68 affected individuals, or in the former members of Yugoslavia (Klarić et al., 2013). While the mycotoxins can enter the body through the skin or the respiratory system, the most common entry point is the gastrointestinal tract (Hedayati et al., 2007). The manifested symptoms depend on the type and form of digested mycotoxins, the amount of intake, the duration of poisoning, age, sex, genetic background, and the health status of the patients (Marroquín-Cardona et al., 2014; Dellafiora et al., 2018; Keller, 2019). The absorption of the different forms of the toxins depends on several factors (Gallo et al., 2015). In the human body, the toxins undergo a detoxification process and may form deposits mostly in the liver, but other tissues could also store them. The mycotoxin derivatives formed *in vivo* in humans and domestic animals may still have pathological effects. As mentioned earlier, mycotoxins can have nephrotoxic, genotoxic, teratogenic, carcinogenic, and cytotoxic properties but are also capable of affecting tumor development due to their antineoplastic potential (Pócsi et al., 2018).

Mycotoxicosis like most types of poisoning can be acute or chronic. Acute poisoning has a rapid onset and characteristic toxicity symptoms, like gastrointestinal discomfort, general malaise and fatigue, or diarrhea due to the damage of the enterocytes. Acute poisoning may occur when large quantities of mycotoxin are consumed in a short period of time. The incidence of acute mycotoxicosis is sporadic. In acute poisoning, the type of mycotoxin exposure can change the mechanism of the disease. The most frequent symptom being acute hepatitis elicited by the toxins. The occurrence of mycotoxin-inflicted hepatitis depends on many factors, e.g., Kwashiorkor, where the resistance of the affected individual to harmful stressors is generally decreased (Shephard, 2008). Other hepatotoxic conditions such as viral hepatitis infections, heavy metals, or alcohol and drug use can propagate the emergence of hepatitis (Saha Turna and Wu, 2019). The chronic mycotoxin poisoning is a worldwide problem. Compared to acute poisoning, the incidence is higher, even so that not all chronic mycotoxicoses are documented. Chronic poisoning is usually a consequence of a low-dose exposure over a long time period, which might result in irreversible effects such as neoplastic diseases (Wu and Santella, 2012; Magnussen and Parsi, 2013). Several factors influence the chronic toxicity of mycotoxins or the occurrence of the first noticeable symptoms. These include the dosage, route of exposure, and the overall health of the affected individual. During chronic mycotoxin exposure, the effects are extensive. The abovementioned basic molecular malfunctions are distinguished but the clinical appearances are varied. The symptoms are slow to appear and hard to connect to a specific disease. This is even more difficult when the mycotoxin exposure is irregular, the nutritional status is not stable, and other factors may alter the overall medical status.

It is not easy to distinguish between acute and chronic toxicities in mycotoxicoses because these diseases can easily be mistaken for other common illnesses with similar symptoms. The current understanding of *Aspergillus*-derived mycotoxins still relies on some case studies (Smith et al., 2016; Udovicki et al., 2018). There are possibilities to measure the mycotoxin levels in the patient’s urine and blood, but without knowing any intake ratio, it is hard to interpret these pieces of information (Escrivá et al., 2017). Although there are some data recorded in larger mycotoxin outbreaks in the third world, any connection between the mycotoxin levels and the severity of the symptoms is difficult to establish. On account of the individual differences, patients with no detectable toxin levels showed symptoms, but these findings could be the consequences of other unrelated diseases. Furthermore, affected individuals with the same mycotoxin urine concentrations had different symptoms (Peraica et al., 1999). There is a well-documented case when a young woman tried, but failed to commit suicide with purified AFB1 (Willis et al., 1980). She took 5.5 mg of AFB1 over 2 days, and a half year later, a total amount of 35 mg in a 2-week period. Different diagnostic methods like X-ray and ultrasound of the liver or urine and blood tests showed no pathological results throughout the years. The lack of symptoms or any other abnormalities in physiological parameters can be explained by her good physical condition and nutritional status.

### Combined Effects of Mycotoxins

Multiple mycotoxicoses may also occur because the human diet is a complex mixture of various ingredients. Simultaneous spoiling of food by more than one toxigenic fungus has been reported many times. Moreover, some fungi are able to produce a broad spectrum of mycotoxins, and it is confirmed that combined physiological effects of mycotoxins are as relevant as the toxicity of a single mycotoxin. The harmful effects of simultaneous exposures to mycotoxins cannot be predicted solely relying on their individual toxicities. Additive, synergistic, or less than additive toxic effects have been proven among different mycotoxins. For example, interactions were shown between OTA, AFs, and their metabolites in a dose-dependent manner, and in lower concentration ranges, their effects were additive. The explanation resides in the fact that both toxins affect DNA pairing and duplication so they could induce carcinogenic malformations. At higher concentrations, the combined effect was less than additive, but it cannot be called antagonistic. The different physiological effects were explained by the fact that AFs and OTA went through the same bioactivation routes by CYP enzymes in the liver; thus, the amount of bioactivated, potent toxin forms was less compared to the separated experiments (Klarić et al., 2013). Combined effects of AFs, OTA, and fumonisins are “hot topics”, but ongoing and future research should put more effort into the combinations of other emerging mycotoxins as well.

In order to understand the combined effects of different mycotoxins, researchers have developed various model systems. Although these experiments are still in their infancy, we aim at presenting some possible methods on how to analyze these effects. Most of the combined mycotoxin tests were done using binary or tertiary systems, and some of them are summarized in Table 3. Intestinal cell lines (e.g., Caco-2 or IPEC-J2) or gastric cell lines (e.g., NCI-N87) are widely used in cytotoxicity and transportation assays because the first host defense barrier against *per os* mycotoxin exposure is the gastrointestinal wall (Wang et al., 2018; Assunção et al., 2019). In order to describe the chronic-combined toxicological effects more accurately, further experimental data are needed, where sub-toxic mycotoxin concentrations should also be tested to simulate real food consumption habits. Obviously, all *in vitro* studies have their own limitations, but a 2- to 3-week-long mycotoxin treatment may represent suitable models of organ-dependent toxicities. Animal models are an efficient alternative to perform toxicity experiments owing to the known genetic background and strictly regulated diet (Alassane-Kpembi et al., 2017).

**TABLE 3:** Some representative combination of different mycotoxins and their interaction types.

**Mycotoxin couples**	**Doses**	**Model system**	**Exposure**	**Interaction type**	**Assays**	**References**
PAT + OTA	PAT: 0.7–100 μM OTA: 1–200 μM	Caco-2 cell line	24 h	Synergism (Lower IC_50_ level) Less than additive (High IC_50_ level)	MTT, TEER	Assunção et al., 2019
AFB1 + OTA	AFB1: 5–25 μM OTA: 2.5–50 μM	Caco-2 cell line and HepG2 cell line	72 h	Synergism and nearly additive (effects were concentration dependent)	MTT	Sobral et al., 2018
STC + OTA	pM to μM	Hep3B cell line	24–48 h	Synergism and Less than additive (Concentration ratio dependent)	MTT, SCE	Anninou et al., 2014
STC + PAT	PAT: 5–30 μM STC: 0–35 μM	*T. pyriformis*	24 h	Synergism and Less than additive (Concentration ratio dependent)	Inhibition of cell proliferation	Mueller et al., 2013
STC + GTX	STC: 0–30 μM GTX: 0–3.5 μM	*T. pyriformis*	24 h	Synergism and Less than additive (Concentration ratio dependent)	Inhibition of cell proliferation	Mueller et al., 2013
PAT + GTX	PAT: 5–30 μM GTX: 0–3.5 μM	*T. pyriformis*	24 h	Synergism	Inhibition of cell proliferation	Mueller et al., 2013
AFB1 + GTX	AFB1: 0.5–128 μg/ml GTX: 2–500 ng/ml	HCE cell line	24–72 h	Synergism	Cell impedance, MTT	Bossou et al., 2017

*TEER, transepithelial electrical resistance; SCE, sister chromatid exchange; MTT, cell viability assay.*

Although AFs, OTA, and FBs are all among those mycotoxins that have been already regulated worldwide, a regulation of the co-occurring different mycotoxins is still missing. This lack of regulations could be explained by several factors. For example, when a foodstuff is deemed to be contaminated by, e.g., AFs, it is not analyzed further, so other contaminations may remain hidden. However, this approach is favorable in terms of food safety and is financially acceptable as well, because the AF-affected food will be discarded anyway. The co-occurrence of different mycotoxins could be the consequence of either pre-harvest or post-harvest technologies. It has been shown that AFs and OTA can be found together mainly in cereals but herbs, spices, and dried fruits are also on the lists of potentially contaminated foods (Almeida et al., 2012; Smith et al., 2016). Furthermore, *A. niger* and *A. carbonarius* have been isolated frequently from grapes grown in Australia, South America, or Europe, and they are responsible for FB2 and OTA content of grape wine (Logrieco et al., 2011; Storari et al., 2012), and these two toxins could be responsible for several neoplastic changes in humans.

However, to set a rational limit for combined mycotoxin exposures, the exact concentrations of co-occurring mycotoxins should be determined, even when the individual concentrations are in the sub-toxic ranges. This will be an important goal for further research in this field because people may consume mycotoxins in sub-toxic concentrations without any detectable symptoms, but the combinations of these sub-toxic exposures may be deleterious (Anninou et al., 2014). An example for chronic-combined effects of mycotoxins could be when they target the same physiological pathways. Complex biological systems, like the immune system, where every aspect of the mechanisms is essential and strictly regulated, are very sensitive to multiple mycotoxin exposures. The production of leukocytes could be impaired due to the genotoxic properties of the mycotoxins and this can decelerate the division of the progenitor cells and, furthermore, the function of the differentiated leukocytes can also be inhibited. As some toxins can negatively affect the protein synthesis of leukocytes, signaling pathways, phagocytosis, and the differentiation of progenitors, the overall result might be a large-scale immunosuppression.

### The Risks of Mycotoxins at Different Stages of Life

Mycotoxicosis can occur at every stages of life, and it can affect the individuals differently according to their age. The harmful effects of mycotoxins on cell division can lead to drastic consequences, which are even more severe during intrauterine life. There are some data on mycotoxicoses in children, infants, and even in embryonic stage, but these topics definitely need additional attention from the scientific community. Using human embryonic stem cells (hESCs), a research group showed the dose dependency of OTA toxicity (Erceg et al., 2019). More data could give us a clearer view on how different mycotoxin exposures affect the differentiation of hESC cells.

**FIGURE 7:**
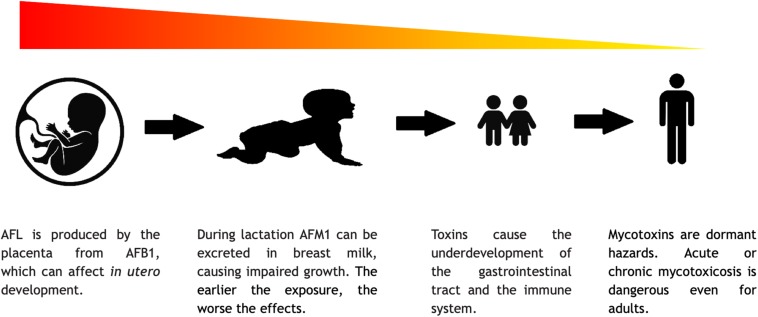
The severity of consequences of mycotoxins in the different stages of life.

Mycotoxins mount variable challenges to humans at different stages of life (Figure 7). They can influence the production of gametes and thus the success of impregnation, because the cytotoxic effect of the mycotoxins could hamper the division and differentiation of the gametes and thus may cause infertility by interfering with, e.g., spermatogenesis (El. Khoury et al., 2019). Mycotoxins can damage the body of the mother, in the abovementioned ways, and can cause nutrition deficit in the embryo, but mycotoxins could also have more direct impacts. AFL, a derivative of AFB1, can go through the placenta and affect the embryo. This phenomenon was already documented in humans, but the adverse effects of this has not yet been fully investigated (IARC, 2015). From animal experiment, it is known that mycotoxins can increase the possibility of stillborn (Kanora and Maes, 2009). During lactation, AFM1 can also be excreted within the breast milk. These circumstances show the additional risks of mycotoxin poisoning in pregnant or breastfeeding women (Ortiz et al., 2018; Rushing and Selim, 2019). According to the above, mycotoxin poisoning is a significant risk to human development. The complex nature of mycotoxicosis can cause various symptoms, which are mostly connected to the cytotoxic and genotoxic properties of the mycotoxins. In newborns and young children, the symptoms may be more severe due to the fact that the mycotoxins like AFs and OTA have a general negative effect on cell multiplication. During development, the lack of adequate cell division may lead to a delay or retention in growth, mental retardation, and severe immunosuppression (Khlangwiset et al., 2011; Watson et al., 2018). *In vitro* studies showed that GTX can alter the connections between astrocytes and neurons, which could affect the formation of the brain and ruin cognitive development (Patel et al., 2018). As fumonisins inhibit sphingolipid biosynthesis, it is cited as a possible cause of neuronal tube defects (Lumsangkul et al., 2019). In children, the not yet fully developed and/or damaged gastrointestinal tract cannot perform its task and the pathways of nutrient absorption are compromised (Herrera et al., 2019). Malnutrition is thus a well-known adverse factor in mycotoxicosis since without sufficient nutrient intake, the body cannot cope with the damage caused by the toxins or any other external factors. Furthermore, the impaired digestive capability makes the treatment even more difficult and less effective (Lombard, 2014). For example, in developed countries, where apple juice is a popular beverage among children, PAT content of such soft drinks should be seriously regulated even though the long-term deleterious physiological effects of PAT have remained yet to be fully understood (Pal et al., 2017). As mentioned earlier, it is hard to distinguish between the effects of malnutrition and mycotoxin exposures, although in the past two decades, governments tried to put effort in a more thorough mycotoxin monitoring. Blood, urine, and maternal milk specimens were mostly analyzed for AFs, OTA, and FB1 (Chen et al., 2018). From these data, the conclusion is that children are typically at high risk under mycotoxin exposures. However, to get a better insight into this matter, an international standard protocol should be introduced (Al-Jaal et al., 2019).

Although healthy adults are endangered as well, their detoxification system can usually handle an acute mycotoxin exposure. However, different environmental factors, like drug abuse, alcoholism, and malnutrition could act as a synergistic factor in mycotoxicoses. In the case of chronic exposures in adults, developmental disorders are not as significant as in childhood. However, tissues where high cell division rate is essential for their function are affected more by the harmful mycotoxins. Hematopoiesis, the function of the enterocytes, or the immune system requires sufficient cell-multiplication where the xenobiotics, like mycotoxins, could have a drastic impact on the cell cycle (Omotayo et al., 2019; Sobral et al., 2019). It is undeniable that the carcinogenic properties of the mycotoxins affect adults as well, and prolonged exposures may cause complex neoplastic diseases (Alshannaq et al., 2017). Throughout the aging process, the adaptive capabilities of the body are decreasing, which could propagate the manifestation of the abovementioned negative effects even earlier. These affected individuals need a complex and life-saving therapy, e.g., liver transplantations or immune therapies, which could take its toll on the global health system.

### Therapeutic Procedures

**FIGURE 8:**
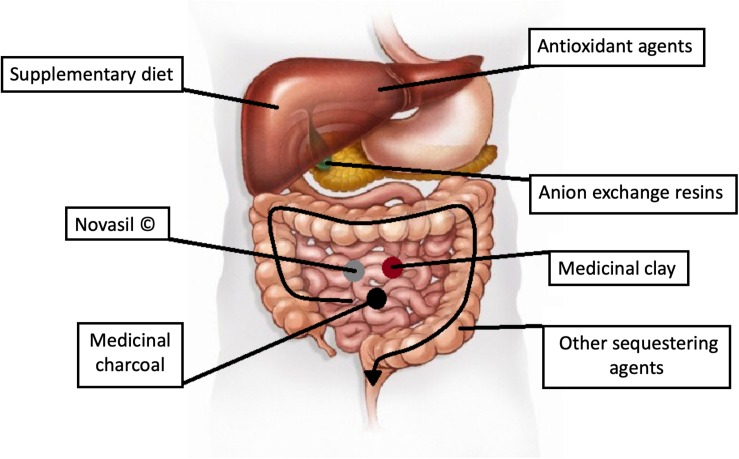
Possible target points for therapy.

It is undeniable that mycotoxicoses represent a serious threat to general health. The symptoms are treatable, although to set appropriate differential diagnosis is quite difficult. When investigating food poisoning, mycotoxins as the underlying factor may usually emerge only when there are no other possibilities left and all other contingencies such as viral gastrointestinal infections or bacterial enterotoxins have been excluded. In acute mycotoxin poisoning, the source of the exposure can easily be recognized as the contaminated foodstuff can be analyzed relatively easily and the given mycotoxin can be identified. However, there are no specific and effective treatments for the different mycotoxicoses until now (Hope, 2013). In the case of acute poisonings, merely the symptoms are usually treated, but these non-specific methods are rarely sufficient. The termination of the exposure to mycotoxins and an appropriate diet could better diminish the symptoms than any other medical procedure. Nevertheless, below, we list the most commonly used current methods to counteract mycotoxin poisonings (see also Figure 8).

Sequestering agents are non-absorbable materials that can bind and neutralize mycotoxins in the gastrointestinal tract (Phillips et al., 2002; Jard et al., 2011). These substances have a large surface-to-volume ratio and, hence, they have a large absorptive capacity. Activated carbon (charcoal) is a non-specific absorbent, and it is a useful agent in multi-mycotoxin poisoning. Clay is a widely studied material, especially in reducing the toxicity of AFs. Novasil^®^ is a frequently studied agent and Phase II clinical trials showed the safety of this material. It has been shown that a 3-month-long treatment decreased the urinary concentrations of AFB1 adducts and reduced the AFM1 level in urine. Other trials were based on Novasil^®^ delivery in capsule, in food, or added to water. Even a daily uptake of 3 g of this absorbent is safe for adults, but a trial in Ghana showed that 0.75 g/day is safe even for children (Hope, 2013; Watson et al., 2018). Cholestyramine (CSM) is an anion exchange resin and acts as a bile sequestering agent. It could reduce the enterohepatic recirculation of fat-soluble mycotoxins. *In vitro* studies showed that CSM has a higher affinity to OTA than to bile salts, and some animal experiments using CSM resulted in decreased plasma and urine levels of OTA but also in an elevated OTA secretion in feces (Hope, 2013).

The boosting of glutathione system may help in the detoxification process. As the detoxification capacity of liver varies with age, sex, and other factors, only boosting this detoxification system is not enough in the neutralization of mycotoxins. Other substances, like Vitamin C, E, D, or Q10 with zinc, could also help to prevent the harmful effects of ROS. Unfortunately, these materials are not specific, and their mechanism of action is based on the reduction of free radicals (Rea et al., 2009; Hope, 2013). Dialysis and other supplementary procedures to aid and protect the affected organs like the liver and bone marrow may also help.

The efficiency of the diagnosis of chronic mycotoxicoses is still low. The symptoms are non-specific and can be easily mistaken for other diseases. Without further clinical investigations, it is thus hard or nearly impossible to differentiate between mycotoxicoses and other diseases. The course of the disease may be modified when Kwashiorkor or other harmful effects such as alcohol are present or the mycotoxin intake is fluctuating. In chronic poisoning, the identification of the different mycotoxins in any feed and food is also difficult, because of the unknown time window of the poisoning. The termination of mycotoxin intake from the food chain and an adequate nutrition can reduce the symptoms considerably in a short period of time. Complementary medical procedures should aid the damaged organs (Yilmaz et al., 2017), and liver or bone marrow transplantation may also be taken into consideration, when the affected organ is completely destroyed. The direct administration of T leukocyte cultures has been hypothesized to have a significant effect (Rea et al., 2009). However, the above mentioned possibilities of treatment are hard to propagate due to their complexity and high costs.

## Concluding Remarks

The presence of mycotoxins in the feed and food chain has been a widespread problem since the beginning of human history. In the past, our possibilities to prevent or treat mycotoxin poisonings were rather limited. Today, with our current knowledge and technical capabilities, we are able to select and use highly efficient, verified methods to mitigate the deleterious effects of mold infestations. However, there are some newly emerging difficulties on the horizon of a mycotoxin free agriculture. The geographical border for harmful mold species like the toxigenic species in the *Aspergillus* genus is moving north as a clear-cut consequence of climate change. The possibility of mold infestations will rise and the size of the affected territory will drastically increase in the near future. To minimize agricultural, economical, and medical risks set by spreading mycotoxins, it will be essential to find a solution for the early detection and the prevention of mycotoxin contaminations. Obviously, there are possibilities to respond to these challenges adequately, but the ideal long-term solution would be a pipeline, which is accepted and followed with independent authorities worldwide to regulate and synchronize the joint community efforts in combating mycotoxins.

Genetically engineered crops could help us to fight off mold infestations even before mycotoxin contaminations have started. Cheap and reliable analytical methods are needed for the early and reliable detection of mycotoxins. The risk of mycotoxins on human health and economy could be neutralized with low-cost detoxification protocols, the implementation of which would require a minimal technical background. The continuous monitoring of storage and processing facilities is also a necessity. The production of foodstuffs with ingredients from different countries should be checked not only in the country where the primary commodity was produced but also in the destination countries once the final product is released into the market. Exposures to multiple mycotoxins may lead to unforeseen toxicological consequences and symptoms, which are currently not known or not investigated yet.

In summary, mycotoxicoses may be a much bigger threat to human health than they currently seem to be. For example, the exact number of people suffering from any kind of acute or chronic mycotoxin poisonings is almost impossible to calculate, and the long-lasting adverse effects of chronic mycotoxin exposures on human health have not been fully realized yet. The carcinogenic effects and developmental disorders might not be the most dangerous features of the *Aspergillus*-derived mycotoxins because they can contribute considerably to human infertility as well. Hence, the impacts of mycotoxins on future generations can be even more significant than we thought before. The increasing frequency of mycotoxin contaminations and the astonishing complexity and variability of multiple mycotoxin exposures might have severe and, at least in part, still hidden effects on public health. Nevertheless, the best time to act should be well before the mycotoxin-related problems have become uncontrollable because prevention is always better and cheaper than to cure an already manifested disease.

## Author Contributions

All authors contributed to writing different sections of the manuscript, worked on compiling the figures, and selecting the appropriate references.

## Conflict of Interest

The authors declare that the research was conducted in the absence of any commercial or financial relationships that could be construed as a potential conflict of interest.
